# Neonatal near Miss and Its Predictors among Neonates Delivered at Debretabor General Hospital, Northern Ethiopia; A Retrospective Analysis

**DOI:** 10.1155/2020/1092479

**Published:** 2020-09-07

**Authors:** Habtamu Abie Tassew, Fisseha Yetwale Kassie, Muhabaw Shumye Mihret

**Affiliations:** ^1^Department of Midwifery, Debretabor University, Debretabor, Ethiopia; ^2^Department of General Midwifery, School of Midwifery, College of Medicine and Health Sciences, University of Gondar, Gondar, Ethiopia; ^3^Department of Clinical Midwifery, School of Midwifery, College of Medicine and Health Sciences, University of Gondar, Gondar, Ethiopia

## Abstract

**Background:**

In many low-resource countries, the progress of neonatal mortality reduction is very slow. The scenario is notably true in sub-Saharan Africa including Ethiopia. For every neonatal death, there are lots of near missed neonates. Generating evidences on the extent and predictors of neonatal near miss is a key step in neonatal mortality reduction efforts. However, there is limited evidence in this aspect in Ethiopia.

**Objective:**

This study is aimed at assessing the proportion of neonatal near miss and associated factors among neonates delivered at Debretabor General Hospital, Northern Ethiopia, 2019.

**Methods:**

An institution-based cross-sectional study was conducted on 422 neonates delivered at Debretabor General Hospital from July 1^st^, 2018, to June 30^th^, 2019. Both pragmatic and management criteria of definition of neonatal near miss were utilized. A systematic random sampling technique was used to select the cards of the study participants. Data were extracted with structured and pretested checklist, entered in the EpiData, and then exported to SPSS version 20. Both descriptive and analytical procedures have been done. Descriptive statistics such as frequencies and cross tabulations were carried out. The binary logistic regression model was fitted and variables with *p* value < 0.20 were entered in the multivariable logistic regression model. Both crude and adjusted odds ratios with the corresponding 95% CI were computed. The level of significance has been claimed based on the adjusted odds ratio with 95% CI and its *p* value of ≤0.05.

**Results:**

The proportion of neonates experiencing near miss was obtained to be 32.2% with 95% CI (28, 36). Rural residence (AOR = 4.41; 95% CI: 2.57,7.55), incomplete ANC visit (AOR = 3.16; 95% CI: 1.90,5.25), primiparous (AOR = 2.55; 95% CI: 1.59,4.12), pregnancy-induced hypertension (AOR = 3.23; 95% CI: 1.19,8.78), premature rupture of membrane (AOR = 4.65; 95% CI: 1.70,12,73), cephalic-pelvic disproportion (AOR = 3.05; 95% CI: 1.32,7.01), and antepartum hemorrhage (AOR = 4.95; 95% CI: 1.89,12.96) were the independent predictors of neonatal near-miss. *Conclusion and Recommendations*. The proportion of neonatal near miss was found to be high in the study setting. Most of the determinants of near miss are modifiable obstetric-related factors. Hence, stakeholders need to consider the aforesaid factors while they design interventions.

## 1. Introduction

Neonatal mortality and morbidity remain a global public health challenge with 99% of the cases happening in middle- and low-income countries (MLIC) [1–4]. Neonatal deaths (ND) account for about 44% of under-five deaths worldwide and 62% of infant deaths in Ethiopia [5, 6]. Despite a remarkable achievement in under-five mortality reduction, the proportion of under-five deaths in the newborn period has increased from 41% to 46% between 2000 and 2016 globally [7]. The scenario is more notable in Ethiopia [8]. For every neonate who dies, there are lots of neonatal near misses (NNM) [5, 6].

Neonatal near miss (NNM) refers to situations where the newborn nearly died between 0 and 28 days of life and they survived either by chance or because of the good quality of care they received [9, 10]. Neonates who survive severe neonatal complications have many characteristics in common with neonatal death events particularly on risk factors. Therefore, achieving investigations on NNM is a more cost-effective approach than conducting population- based study on neonatal death since the latter one requires a long time and huge resources. In addition, the study will contribute for the implementation of target two of Sustainable Development Goal (SDG) 3, which is planned to end preventable deaths of newborns and children under 5 years of age with all countries aiming to reduce neonatal mortality to at least as low as 12 per 1,000 live births and under-5 mortality to at least as low as 25 per 1,000 live births by 2030 [11].

Despite this fact, there was scarce of evidence in this aspect in Ethiopia in general. Even the antecedently identified risk factors of NNM such as maternal hypertension, low birth weight, and prematurity could vary across settings and time trends [12, 13]. By taking these points of views into account, we have conducted the current study which is aimed at assessing the proportion of NNM and associated factors among neonates delivered at Debretabor General Hospital, Northern Ethiopia, 2019.

## 2. Methods

An institutional-based retrospective cross-sectional study was conducted at Debretabor General Hospital (DGH) from July 1^st^, 2018, to June 30^th^, 2019. DGH is found in Debretabor town, South Gondar zone, Amhara national regional state, Northern Ethiopia. Debretabor town is located about 665 kilometers away from Addis Ababa—the capital city of Ethiopia.

Debretabor General Hospital (DGH) provides health services to more than 2 million population. In its catchment area, there are seven primary Hospitals and 93 health centres. The Hospital offers by average about 8136 inpatient services through five wards, namely, Medical, pediatrics, Neonatal Intensive Care Unit (NICU), Gynecology/Obstetric, and Surgery and Ophthalmology ward. In the Gynecology/Obstetric ward, there are 27 Midwives, 8 Gynecologist, and 5 integrated emergency surgical officer. A total of 3720 deliveries were reported in the last one year [14].

All live birth neonates who delivered in DGH from July 1^st^, 2018, to June 30^th^, 2019, were included in the study.

The sample size was determined by using single population proportion formula: *n* = (*Z* *a*/2)^2^ *P* (1 − *P*)/*W*^2^ where *n*=total sample size, *Z*=confidence interval (with 95% level of certainty), *w*=margin of error (5%), and *p*=proportion of live born neonates surviving from life-threatening conditions (50% since there was no previous study). Thus, *n* = (1.96)^2^ × 0.5 × 0.5/(0.05)^2^ = 384. By adding 10% of the nonresponse rate, the final sample size is turned to be 422.

Maternal and neonatal cards were selected by using the systematic random sampling technique based on the registration order. A total of 3720 live births were found to be documented in the registration log book during the study period. A *K*^th^ interval was made by dividing the annual last year report to the calculated sample size (i.e., 3720/422) and approximates to be 9. Thus, a total of 422 cards of the neonates and the mothers were selected and reviewed. On the other hand, neonatal charts which had no discharge summaries were planned to be excluded. However, there was no chart excluded based on this criterion.

The outcome variable of the study is neonatal near miss (NNM). We used both pragmatic and management criteria of definition of NNM [10]. Accordingly, NNM was considered when the neonate met at least one of the following criteria: birth weight < 1750 grams, gestational age < 33 weeks, 5^th^-minute Apgar score < 7, new born resuscitated with bag and mask, cardiopulmonary resuscitation (CPR), intubation, nasal continuous positive airway pressure (NCPAP), use of parenteral antibiotics, parenteral nutrition, vasoactive drugs, phototherapy, anticonvulsants, and blood products or steroids [10, 15]. The explanatory variables include maternal age, residence, parity, antenatal care (ANC) visit, previous still birth, previous caesarean section, premature rupture of membrane (PROM), mode of delivery, pregnancy-induced hypertension (PIH), antepartum hemorrhage (APH), cephalopelvic disproportion (CPD), low birth weight, Gestational age (GA), multiple deliveries, hypertension, diabetes mellitus, anemia, HIV status, history of abortion, and sexually transmitted infection (STI).

Prior to the commencement of data collection, we have obtained an ethical clearance from the Institutional Review Board of University of Gondar and letter of cooperation from Medical director office of Debretabor General Hospital. Thereafter, the data were extracted through chart review using pretested structured check lists. A one-day training was provided to the data collectors and the supervisors. Pretest was done on 21 individuals (i.e., 5% of the total sample size). After selection and search of cards, data were extracted from the records in the cards by three trained BSc midwives and supervised by two MSc midwives. In case of twin deliveries, one of the babies was selected randomly. Throughout the course of the data collection, data collectors have been supervised, and regular meetings were held to address any issues. The collected data were reviewed and checked for completeness before data entry. Data were then entered in the EpiData and exported to SPSS version 20. Both descriptive and analytical procedures have been done. Descriptive statistics such as frequencies and cross tabulations were then carried out, and the results were presented in texts and tables. The binary logistic regression model has been fitted in the analytical procedure. Initially, bivariable analysis was done for all explanatory variables in relation to the NNM and variables with *p* value < 0.20 in the bivariable analysis were candidates to be entered in the multivariable logistic regression model. Both crude and adjusted odds ratios with the corresponding 95% CI were computed. The level of statistical significance was claimed based on the adjusted odds ratio with 95% CI and its *p* value of ≤0.05.

## 3. Results

### 3.1. Sociodemographic Characteristics

**Figure 1:**
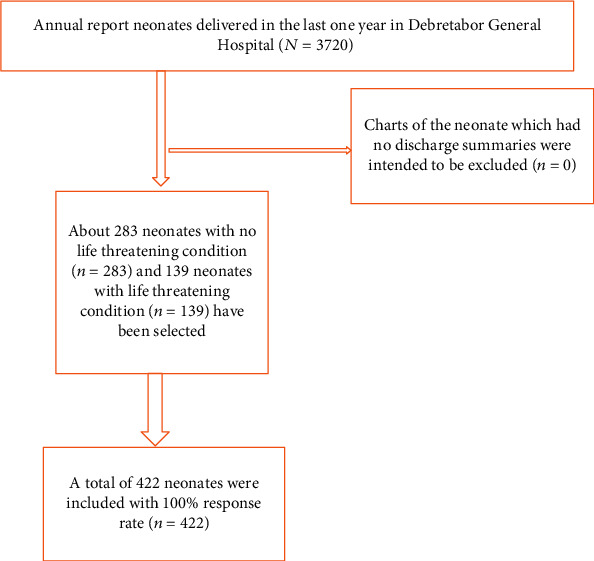
STROBE flow chart illustrating the inclusion of the neonates in the study neonates delivered at Debretabor General Hospital, Northern Ethiopia, 2019 (*n* = 422).

A total of 422 mother's and neonate's cards were reviewed with a response rate of 100% (Figure 1). Of the sociodemograhic variables, only age and residence were obtained completely in the document. Accordingly, about 288 (68.2%) of the mothers' age lay in the age group of 20-34 years with the mean age (±SD) of 28.4 years (±6.5 SD), whereas, about 102 (24.2%) and 32 (7.6%) of the mothers' age were documented to be within 35-49 years and 18-20 years, respectively. Majority (78.2%) of the mothers were recorded to be urban residents.

### 3.2. Obstetrics/Gynecology- and Medical-Related Characteristics

All mothers of newborns had at least one ANC visit, and about 118 (28%) of the mothers had incomplete (<4) ANC visit. About 123 (29.1%) and 27 (16.4%) of **t**he mothers experienced obstetric complications and anemia, respectively, during the indexed pregnancy (Table 1).

### 3.3. Proportion of Neonatal Near Miss

The proportion of NNM among live birth neonates delivered at Debretabor General Hospital was found to be 32.9% with 95% CI (28, 36). More than half (58%) of the NNM cases faced more than one NNM criteria. About 126 (90.7%), 123 (88.5%), and 112 (80.6%) of the near-missed neonates were resuscitated with a bag and mask, cardiopulmonary resuscitation, and continuous positive airway pressure, respectively. Similarly, about 117 (84.2%) of the near-missed neonates were treated with parenteral antibiotics (Table 2).

### 3.4. Predictors of Neonatal Near Miss

Certain variables including maternal age, history of abortion, history of still birth, history of diabetes mellitus, mode of delivery, maternal anemia, history of STI, rural residence, incomplete ANC visit, parity, PROM, PIH, APH, and CPD were associated with NNM in the bivariate analysis, whereas rural residence, incomplete ANC visits, primiparous, PROM, PIH, APH, and CPD had statistical significant association with NNM in the multivariable analysis.

Accordingly, the odds of NNM were higher among neonates born to mothers who had incomplete ANC visit (AOR = 3.16; 95% CI: 1.90, 5.25), resided in a rural area (AOR = 4.41; 95% CI: 2.57, 7.55), was primiparous (AOR = 2.55; 95% CI: 1.59, 4.12), had PROM (AOR = 4.65; 95% CI: 1.70, 12.73), had PIH (AOR = 3.23; 95% CI: 1.19, 8.78), had APH (AOR = 4.95; 95% CI: 1.89, 12.96), and had CPD (AOR = 3.05; 95% CI: 1.32, 7.01) in the indexed pregnancy as compared to their counterparts (Table 3).

## 4. Discussion

This study assessed the proportion and associated factors of NNM among neonates delivered at Debretabor General Hospital during the last one year. The current study revealed that the proportion of NNM was high and it was independently predicted by incomplete ANC visit, rural residence, primiparous, PROM, PIH, APH, and CPD.

In the current study, nearly one in three (32.9%) neonates experienced NNM. This finding is in line with the study done in Uganda—36.7% [16]. The agreement in magnitude across the two studies might be due to the fact that both of the studies are conducted in poor resource settings, where neonatal morbidities or mortalities tend to be higher.

However, the proportion of NNM in the current study is higher than that of the magnitude reported in northeast Brazil 22% [17], WHO multicounty survey finding 7.25% [18], Brazil survey 3.3% [19], and southeast Brazil 1.7% [20]. This discrepancy might be due to the variation in NNM definition. Moreover, the inconsistency in the magnitude of NNM could be ascribed to the difference in study settings. Most of the aforementioned studies had been done at the study settings with good health care systems where neonatal morbidities (near misses) tend to be low, unlike the current one.

In this study, about 126 (90.7%), 123 (88.5%), and 112 (80.6%) of the near-missed neonates were resuscitated with a bag and mask, cardiopulmonary resuscitation, and continuous positive airway pressure, respectively. These observed high frequencies might be because of the current study being conducted in a hospital center of women with high-risk pregnancies and high flow of referral cases; this might be the source of bias that resulted in high frequencies of the observed NNM criteria. Likewise, the use of a nasal CPAP device was the most commonly identified criterion reported on a study done in Northeastern Brazil [17] with a frequency of 62.9% which was found to be relatively lower when compared to the present study. This might be due to the discrepancy in sociodemograhic characteristics and a disparity in the quality of health care delivery system among the study participants of the studies. Mothers in the poor resource counties are believed to have delayed health seeking behaviors to pregnancy, labor, and delivery complications and this would contribute to the high prevalence of NNM with the use of advanced neonatal resuscitation procedures by the skilled birth attendants like nasal CPAP in order to help babies breathe during deliveries.

The odds of experiencing NNM were four times higher among neonates born to rural resident mothers than their counterparts. This could be explained by the disparity in accesses to health care service and health information across residences. In most Ethiopian rural areas, the distribution of health facilities is far scattered. This might contribute for delay in receiving appropriate emergency, obstetric, and neonatal health care services. This finding reminds the stakeholders to oversee the health care system distribution in the rural areas.

Empirical evidences showcase that the neonatal and the maternal health outcomes magnanimously vary across parity. The current analysis has presented with a similar finding. Accordingly, neonates born to primiparous women were 2.55 times more likely to experience NNM as compared to their counterpart. This finding is also supported by a previous study done in Southeast Brazil [20]. This might be due to the fact that primiparous women tend to pose fewer experiences in identification of danger signs and respective measures during pregnancy and child birth.

Existing studies corroborate that incomplete ANC visits have been linked to poor pregnancy outcomes [21, 22]. Similarly, the current analysis exhibited that incomplete ANC visit is an independent determinant of NNM. Hence, neonates born to mothers who had incomplete ANC visits were 3 times more likely to face NNM as compared to their congruent. This finding is in accordance with that of the previous studies reported in Ethiopia and Southeast Brazil [23, 24]. This might be explained by the fact that incomplete ANC visit procreates inadequate care provision during pregnancy and it influences the maternal continuum of care thereby affects the neonatal health outcomes.

Premature rupture of membrane (PROM) is documented to be a risk factor for poor neonatal health outcomes since it predisposes neonates for plenty of complications such as sepsis [25, 26]. According to the current study, neonates born to women who had PROM in the indexed pregnancy were 4.65 times more likely to encounter NNM as compared to the counter parts. This is consistent with the study's finding done previously in Ethiopia [23].

This study signifies that the odds of experiencing NNM were 4.95 times higher among neonates born to women who had APH in the recent pregnancy than their counters. The finding is congruent with the study done in Zimbabwe and Ethiopia [23, 27]. This is due to the fact that fetal circulation is placental dependent and thus APH compromises the fetal blood perfusion which in turn sorts to neonatal morbidities.

Likewise, neonates born to mothers who had PIH in the indexed pregnancy were 3 times more probably to be near missed as compared to their congruent. The result is in line with the study done in Zimbabwe [27]. It is also in agreement with previous studies [28, 29]. The finding advocates that hypertension in pregnancy constitutes a major health burden in the obstetric population due to its effect on uteroplacental insufficiency and vital organ damage [30].

This study has demonstrated that NNM is independently predicted by CPD. Thus, neonates born to mothers with CPD were 3 times more likely to encounter NNM as compared to those neonates delivered to women with no CPD. The finding is in alignment with previous study's finding executed in Ethiopia at Jimma University Specialized Hospital [29]. The finding might be ascribed to the effect of CPD on prolonged labor which in turn causes neonatal complications.

Generally, as there was a scarce of evidence regarding NNM in Ethiopia, the result of the current study will be used as a baseline finding for the incoming researchers, policy makers, and/or anybody concerned on neonatal health issues. Moreover, the finding of this study can be representative as it utilized the probable sampling technique with adequate sample sizes. However, it had its own limitations too. Since it used a secondary data, there was difficulty of getting some important variables such as maternal educational level. These variables could give readers a clue for interpretations of the result.

## 5. Conclusions

The proportion of neonatal near miss in the study setting was high. Rural residence, incomplete ANC visit, primiparous, PIH, PROM, APH, and CPD were its independent predictors. Thus, preventions, early detections, and timely treatments of obstetric complications, such as PIH, PROM, APH and CPD, would decrease the burden of NNM. Moreover, strengthening awareness creations on the importance of full focused ANC attendance, expanding the quality health care services to rural areas, and offering tailored obstetric counseling for pregnant women more particularly primigravidas have been recommended.

## Figures and Tables

**Table 1:** Obstetrics/gynecology- and medical-related characteristics of the participants in Debretabor General Hospital, Northern Ethiopia, 2019 (*n* = 422).

Variables	Number	Percent
ANC visit		
1-3	118	28
≥4	304	72
Parity		
Primiparous	182	43.1
Multiparous	240	56.9
History of abortion		
Yes	101	23.9
No	321	76.1
Mode of delivery		
Caesarean delivery	64	15.2
Vaginal delivery	358	84.8
History of STI		
Yes	12	2.8
No	410	97.2
Maternal anemia		
Yes	27	16.4
No	395	83.6
History of hypertension		
Yes	61	14.5
No	361	85.5
History of DM		
Yes	20	4.7
No	402	95.3
Sero status of the mother		
Reactive	3	0.7
Nonreactive	419	99.3

**Table 2:** Neonatal near miss across measurement criteria among near-missed neonates in Debretabor General Hospital, Northern Ethiopia, 2019 (*n* = 139).

Criteria	Number (percent)
Gestational age less than 33 completed weeks	19 (13.7)
Weight less than 1.7 kg	82 (59.0)
5-minute APGAR score < 7	71 (51.1)
Resuscitated with bag and mask	126 (90.7)
Cardiopulmonary resuscitation (CPR)	112 (82.4)
Intubation	44 (31.7)
Nasal continuous positive airway pressure	123 (88.5)
Parenteral antibiotics	117 (84.2)
Use of parenteral nutrition	40 (28.8)
Vasoactive drugs	15 (10.8)
Phototherapy during the first 28 days	87 (62.6)
Use of blood product	22 (15.8)
Anticonvulsants	8 (5.8)
Use of steroids for refractory hypoglycemia treatment	1 (0.7)

**Table 3:** Predictors of neonatal near miss among neonates delivered at Debretabor General Hospital, Northern Ethiopia, 2019 (*n* = 422).

Variables	NNM		COR (95% CI)	AOR (95% CI)
	Yes	No		
Age				
<20	16	16	2.27 (1.08, 4.75)	2.05 (0.90, 4.72)^∗^
20-34	88	200	2.19 (0.97, 4.91)	2.41 (0.93, 6.23)^∗^
35-49	32	70	1	1
Residence				
Urban	83	247	1	1
Rural	53	39	4.04 (2.50, 6.55)	**4.41 (2.57, 7.55)**∗∗
Number of ANC				
1-3	58	60	2.80 (1.80, 4.36)	**3.16 (1.90, 5.25)**∗∗
≥4	78	226	1	1
History of abortion				
Yes	43	70	1.43 (0.91, 2.24)	1.26 (0.69, 2.28)∗
No	93	216	1	1
Parity				
Primiparous	79	103	2.46 (1.62, 3.74)	**2.55 (1.59, 4.12)**∗∗
Multiparous	57	183	1	1
History of still birth				
Yes	18	21	1.92 (0.99, 3.75)	1.95 (0.87, 4.39)∗
No	118	265	1	1
History of DM				
Yes	11	9	2.71 (1.10, 6.70)	1.21 (0.38, 3.87)∗
No	125	277	1	1
Mode of delivery				
C/S delivery	26	38	1.54 (0.89, 2.66)	1.23 (0.63, 2.40)∗
Vaginal delivery	110	248	1	1
Maternal anemia				
Yes	12	15	1.75 (0.79, 3.85)	1.24 (0.46, 3.34)∗
No	124	271	1	1
PROM				
Yes	14	8	3.99 (1.63, 9.75)	**4.65 (1.70, 12.73)**∗∗
No	122	278	1	1
STI				
Yes	7	5	3.05 (0.95, 9.79)	2.02 (0.50, 8.17)∗
No	129	281	1	1
PIH				
Yes	17	9	4.40 (1.91, 10.14)	**3.23 (1.19, 8.78)**∗∗
No	119	277	1	**1**
APH				
Yes No	15 121	10 276	3.42 (1.50, 7.83) 1	**4.95 (1.89, 12.96)**∗∗ 1
CPD				
Yes	25	14	4.38 (2.19, 8.73)	**3.05 (1.32, 7.01)**∗∗
No	111	272	1	1

## Data Availability

The datasets employed in the current study can be available from the corresponding author upon the reasonable request.

## Abbreviations

APH: Antepartum hemorrhage
AOR: Adjusted odds ratio
COR: Crude odds ratio
C/S: Caesarean section
CPD: Cephalo pelvic disproportion
DM: Diabetes mellitus
NICU: Neonatal intensive care unit
NNM: Neonatal near miss
PIH: Pregnancy induced hypertension
PROM: Premature rupture of membrane
SPSS: Statistical package for science solution
STI: Sexually transmitted infection
SVD: Spontaneous vertex deliver
WHO: World Health Organization.

## References

[1] Tran H. T., Doyle L. W., Leed K. J., Grahamb S. M. (2012). A systematic review of the burden of neonatal mortality and morbidity in the ASEAN Region. *WHO South-East Asia Journal of Public Health*.

[2] Liu L., Oza S., Hogan D. (2015). Global, regional, and national causes of child mortality in 2000–13, with projections to inform post-2015 priorities: an updated systematic analysis. *The Lancet*.

[3] WHO (2015). *Sustainable development goal 3*.

[4] Sankar M. J., Natarajan C. K., Das R. R., Agarwal R., Chandrasekaran A., Paul V. K. (2016). When do newborns die? A systematic review of timing of overall and cause-specific neonatal deaths in developing countries. *Journal of Perinatology*.

[5] Wardlaw T., You D., Newby H., Anthony D., Chopra M. (2013). Child survival: a message of hope but a call for renewed commitment in UNICEF report. *Reproductive Health*.

[6] Wardlaw T., You D., Hug L., Amouzou A., Newby H. (2014). UNICEF Report: enormous progress in child survival but greater focus on newborns urgently needed. *Reproductive Health*.

[7] Ezeh O. K. (2017). Trends and population-attributable risk estimates for predictors of early neonatal mortality in Nigeria, 2003–2013: a cross-sectional analysis. *BMJ Open*.

[8] WHO (2014). *Millinium Development Goal Report*.

[9] Pileggi C., Souza J. P., Cecatti J. G., Faundes A. (2010). Neonatal near miss approach in the 2005 WHO Global Survey Brazil. *Jornal de Pediatria*.

[10] Santos J. P., Cecatti J. G., Serruya S. J. (2015). Neonatal near miss: the need for a standard definition and appropriate criteria and the rationale for a prospective surveillance system. *Clinics*.

[11] United Nations (2015). *Sustainable development goals*.

[12] Katz J., West KP Jr, Khatry S. K. (2003). Risk factors for early infant mortality in Sarlahi district, Nepal. *Bulletin of the World Health Organization*.

[13] Mason E., McDougall L., Lawn J. E. (2014). From evidence to action to deliver a healthy start for the next generation. *Lancet*.

[14] (2018). Debre Tabore General Hospital Plan and programme office.

[15] Kale P. L., Jorge M., Laurenti R., Fonseca S. C., Silva K. S. D. (2017). Pragmatic criteria of the definition of neonatal near miss: a comparative study. *Revista de Saúde Pública*.

[16] Nakimuli A., Mbalinda S. N., Nabirye R. C. (2015). Still births, neonatal deaths and neonatal near miss cases attributable to severe obstetric complications: a prospective cohort study in two referral hospitals in Uganda. *BMC Central*.

[17] de Lima T. H. B., Katz L., Kassar S. B., Amorim M. M. (2018). Neonatal near miss determinants at a maternity hospital for high-risk pregnancy in Northeastern Brazil: a prospective study. *BMC Pregnancy and Childbirth*.

[18] Pileggi-Castro C., Camelo Jr J. S., Perdoná G. C. (2014). Development of criteria for identifying neonatal near-miss cases: analysis of two WHO multicountry cross-sectional studies. *BJOG : an International Journal of Obstetrics and Gynaecology*.

[19] Silva A. A. M. D., Leite Á. J. M., Lamy Z. C. (2014). Neonatal near miss in the birth in Brazil survey. *Cadernos de saude publica*.

[20] Kale P. L., Mello-Jorge M. H. P., Silva K. S. D., Fonseca S. C. (2017). Neonatal near miss and mortality: factors associated with life-threatening conditions in newborns at six public maternity hospitals in Southeast Brazil. *Cadernos de saude publica*.

[21] Wondemagegn A. T., Alebel A., Tesema C., Abie W. (2018). The effect of antenatal care follow-up on neonatal health outcomes: a systematic review and meta-analysis. *Public Health Reviews*.

[22] Haftu A., Hagos H., Mehari M.-A. B., G/her B. (2018). Pregnant women adherence level to antenatal care visit and its effect on perinatal outcome among mothers in Tigray Public Health institutions, 2017: cohort study. *BMC Research Notes*.

[23] Mersha A., Bante A., Shibiru S. (2019). Factors associated with neonatal near-miss in selected hospitals of Gamo and Gofa zones, southern Ethiopia: nested case-control study. *BMC Pregnancy and Childbirth*.

[24] Santos J. P., Pileggi-Castro C., Camelo J. S. (2015). Neonatal near miss: a systematic review. *BMC Pregnancy and Childbirth*.

[25] Plucinska A., Hajduczenia M., Pastusiak M., Kowalik M., Miechowicz I., Szymankiewicz M. (2010). The impact of premature rupture of membranes (PROM) on neonatal outcome. *Ginekologia Polska*.

[26] Merenstein G. B., Weisman L. E. (1996). Premature rupture of the membranes: neonatal consequences. *Seminars in Perinatology*.

[27] Muti M. T. M., Tshimanga M., Notion G. T., Bangure D., Chonzi P. (2015). Prevalence of pregnancy induced hypertension and pregnancy outcomes among women seeking maternity services in Harare, Zimbabwe. *BMC Cardiovascular Disorders*.

[28] Cherie N., Mebratu A. (2017). Adverse Birth Out Comes and Associated Factors among Delivered Mothers in Dessie Referral Hospital, North East Ethiopia. *Nursing and Health Care*.

[29] Yeshialem E. A. M., Tesfay A. (2019). Determinants of adverse pregnancy outcomes among mothers who gave birth fromJan 1- DEC31/2015 in Jimma University Specialized Hospital,case control study,2016. *Ethiopian Journal of Reproductive Health*.

[30] Okoye H. C., Eweputanna L. I., Korubo K. I., Ejele O. A. (2016). Effects of maternal hypertension on the neonatal haemogram in southern Nigeria: a case-control study. *Malawi Medical Journal : the Journal of Medical Association of Malawi*.

